# Lose Sleep, Gain Weight: Another Piece of the Obesity Puzzle

**DOI:** 10.1289/ehp.118-a28

**Published:** 2010-01

**Authors:** Angela Spivey

**Affiliations:** **Angela Spivey** writes from North Carolina about science, medicine, and higher education. She has written for *EHP* since 2001 and is a member of the National Association of Science Writers

It’s 11 p.m., and you sit in front of a glowing computer screen, writing e-mails and eating a sandwich. You’ll work until after midnight, when you’ll fall asleep in front of the light and blare of a TV before rising again at 6 a.m. What’s wrong with this picture? Because of modern conveniences and pressures, many of us keep our bodies exposed to light, food, and activity at times when our organs and cells expect dark, quiet, and sleep.

In epidemiologic studies, shorter sleep has been correlated with incidence of obesity, hypertension, and other metabolic disorders. Experimental sleep studies find a similar connection. Increasingly, studies of the possible mechanisms behind these associations suggest that lack of sleep is part of a bigger problem with the 24/7 lifestyle many people today lead. Increasingly, scientists are finding that many physiologic activities related to metabolism don’t happen continuously but oscillate on a regular schedule. Studies in mice as well as humans suggest that when our internal clock is disrupted, it may throw off many bodily functions, especially metabolism.

Many environmental factors have been shown to contribute to circadian disruption. Noise in busy hospitals, street noise, and airport noise have all been reported to disrupt sleep or reduce its quality. Research in animals and humans shows that exposure to light during early biological night resets the main circadian clock by producing a phase delay (the biological urge to go to sleep and wake up later than usual), and exposure during late biological night results in a phase advance (going to sleep and waking up earlier than usual).

We live in a world where air passengers can see the glow of major cities 200 miles away. So the fact that human circadian systems appear sensitive even to low-level artificial light exposure raises significant concerns for the health effects of our electrified modern society. For instance, exposure to a few hours of ordinary room light of about 100 lux brightness (which most people get every night before they go to bed) can significantly reset the human circadian pacemaker, Jamie Zeitzer and colleagues reported in the September 2005 *American Journal of Physiology—Regulatory, Integrative and Comparative Physiology*. However, it’s not certain what the effects of very brief light exposures may be. The duration of light exposure needed to cause shifts hasn’t been well studied, according to a review by Charles Czeisler and Joshua Gooley in volume 27 (2007) of *Cold Spring Harbor Symposia on Quantitative Biology*. [For more information about the health effects of too much artificial light, see “Missing the Dark: Health Effects of Light Pollution,” *EHP* 117:A20–A27 (2009).]

Epidemiologic studies suggest that lack of sleep or sleeping on an altered schedule is an independent risk factor for gaining weight. But it’s still not certain whether short sleep actually causes obesity and its associated health effects. For instance, some scientists have suggested that the association between obesity and lack of sleep may be due to the fact that people who are obese may be more likely to have a sleep disorder such as sleep apnea, or that the reported lack of sleep is a symptom of psychosocial stress. But a body of studies have shown a connection between short sleep and obesity, other health effects associated with obesity, and increased appetite or food intake.

## The Obesity Connection

In one such study, Sanjay R. Patel and colleagues analyzed data from the Nurses’ Health Study and found that women who reported sleeping 5 or fewer hours per night were at greater risk for weight gain and in general weighed more compared with women who slept 7–8 hours per night. These findings appeared in the 15 November 2006 *American Journal of Epidemiology*. Because this type of study relies on self-report of amount of time slept—which people tend to overestimate, according to a study by Diane Lauderdale and colleagues in the 1 July 2006 *American Journal of Epidemiology*—the magnitude of the effect may actually be greater than estimated.

Other epidemiologic studies have measured actual sleep time with wrist actigraphy, which involves attaching an instrument that measures physical movement to participants’ wrists and using lack of wrist movement as an indicator of actual time slept. These studies also showed a link between reduced sleep and obesity, with weight gain attributed to increases in fat, not muscle mass. Sleep apnea was ruled out as a cause for the association through the use of recordings of brain waves and other physical measurements (polysomnography) in a portion of the study participants.

In a November 2009 special issue of *Obesity Reviews* devoted to the role of circadian biology in obesity and metabolism, sleep epidemiologist Jim Gangwisch of the University of Columbia and colleagues also pointed to the need to explore how quality of sleep affects obesity. Some studies have shown, for instance, that it may not be the total sleep time that matters but how much time is spent in the various stages of sleep—in other words, your sleep architecture. In the 1 April 2009 issue of *Sleep*, Madhu Rao and colleagues reported using polysomnography to find that men who got less slow-wave sleep (a stage considered the deepest sleep, which occurs just before “rapid eye movement” sleep) were more likely to currently be obese, even after controlling for total sleep time.

Several studies have linked weight gain associated with short sleep to changes in appetite-regulating hormones such as leptin and ghrelin. Among this work are reports from Karine Spiegel and colleagues in the 7 December 2004 *Annals of Internal Medicine*, and from Shahrad Taheri and colleagues in the December 2004 *PLoS Medicine*. But a study led by University of Chicago endocrinologist Plamen Penev that tried to approximate long-term sleep deprivation in everyday life suggests the relationship between sleep and appetite regulation may be somewhat complex.

As they reported in the January 2009 *American Journal of Clinical Nutrition*, Penev and colleagues had 11 middle-aged, sedentary participants slumber in a controlled sleep laboratory, sleeping fewer than 5.5 hours a night for one 14-day period and more than 7 hours a night during a different 14-day period. The order of the sleep periods (whether the short-sleep period or the normal-sleep period came first) was randomized. The participants were served meals made up of foods they reported eating at home, and they had free access to snacks. “The food they received was served in excess so they could determine their portion sizes themselves,” Penev says.

During the sleep-deprived period, the participants ate more calories—mostly from carbohydrate-rich snacks rather than meals—but their leptin and ghrelin levels did not change. In contrast, participants in previous studies were fed a controlled amount of calories via glucose infusion, and that may have made the difference, Penev says.

“I think the changes in leptin and ghrelin in controlled laboratory experiments have been seen mostly at times when the food intake of participants has been limited or mildly restricted,” Penev says. “But when the subjects have recently consumed excess calories, then sleep loss does not seem to trigger those changes.” Still, the effects on food intake in Penev’s study, although modest, were enough to cause increased weight gain in the long run. Larger and longer studies are needed to confirm these findings, he says.

Other preliminary findings from Penev’s research group, presented in an abstract at the 2009 meeting of the Associated Professional Sleep Societies, suggests lack of sleep may make it harder to lose fat. In a small experimental study, people on a reduced-calorie, nutritionally balanced diet were sleep-restricted to fewer than 5.5 hours for one 14-day period and in a separate 14-day period were allowed to sleep more than 7 hours a night. The two study periods were several months apart, and again, their order was randomized. The participants lost similar amounts of weight during the two periods, but during sleep restriction, fat made up only 26% of the weight loss, while during the normal sleep period fat made up 57% of the weight loss.

## More than Just Gaining Weight

Many epidemiologic and experimental studies link short or disrupted sleep to elements of one of the major health problems linked to obesity: metabolic syndrome, which includes a variety of symptoms that can lead to heart disease, stroke, or diabetes, including high triglycerides and cholesterol, hypertension, insulin resistance, and glucose intolerance.

In a November 2001 study in *Occupational and Environmental Medicine*, Berndt Karlsson of University Hospital in Sweden analyzed data from a study of more than 27,000 workers and found that high triglycerides and low concentrations of high-density lipoprotein (“good”) cholesterol seemed to occur more often in shift workers than in day workers. People with restricted sleep in experimental studies have also shown increased blood pressure as well as increased excretion of noradrenaline in the urine. These changes suggest increased activity of the sympathetic nervous system, which in general raises heart rate and blood pressure.

In addition, Gangwisch suggests short sleep can contribute to hypertension by disrupting the normal nightly decrease in blood pressure. “When we sleep, our blood pressure dips by ten to twenty percent,” he says. “So the less we sleep, the higher our average twenty-four-hour blood pressure is going to be, and over time that can entrain our blood pressure to operate at a higher equilibrium.”

Sleep has also been shown to influence how the body uses insulin and processes glucose. In Penev’s study of middle-aged adults with self-determined consumption of meals and snacks, at the end of the sleep-deprived periods the participants showed increased insulin resistance and decreased glucose tolerance, as reported in the September 2009 *Journal of Clinical Endocrinology and Metabolism*.

Previous studies have shown this effect from short-term severe sleep restriction (sleeping fewer than 4 hours a night), but this study was the first to show it with more prolonged and milder sleep restriction, which is more likely to occur in everyday life. If experimental studies continue to link lack of sleep with elements of metabolic syndrome, a larger-scale interventional study may be in order, some researchers suggest.

“Lack of sleep is just one of many risk factors for metabolic syndrome,” Gangwisch says. “All things being equal, would improving sleep . . . help reverse the condition? Presumably it would, based upon the evidence we have, but that question needs to be studied.” Experimental studies have been of short duration, but Penev says following a group for months or years after a behavioral intervention to reduce chronic loss of sleep may be informative.

## Summer All the Time

In making conceptual links between obesity and short sleep, some scientists point to the idea that our body clocks evolved to fit the lifestyles of our hunter–gatherer ancestors, who had no artificial lighting. They may have slept less in the longer days of summer, which was also the time for storing up fat reserves for the lean winter ahead. But today’s common sleep-deprived, electrified modern lifestyle, combined with readily available food year-round, may be telling our internal clocks it’s summer all the time. “The short sleep durations could be a signal to our metabolic regulatory systems that it’s summertime—it’s time to go out, gain weight, build up fat reserves, to prepare for winter,” says Gangwisch.

That sounds logical, but to really test the idea, scientists study the mechanisms of the body’s circadian clock, which is run not by gears and springs but by a set of positive and negative transcription factor feedback loops that regulate the expression of themselves as well as other downstream genes. “As the amounts of the positive factors rise, they stimulate expression of the . . . proteins that will ‘sit on top’ and inhibit the positive factors’ expression,” says Molly Bray, a molecular geneticist at the University of Alabama at Birmingham. “As the positive loop gets inhibited, then of course the negative factors decrease because there is nothing to stimulate their production.”

This process cycles every 24 hours. Some of the genes known to be involved in these loops include *CLOCK*, *BMAL1*, and *PER1* and *PER2*. Mouse models in which these genes are mutated in all body tissues exhibit disruptions in their eating, sleep, and metabolic functions, highlighting the role of the circadian clock in metabolism and obesity.

When most people speak of the circadian clock, they think of the central clock in the brain, located in the suprachiasmatic nucleus of the hypothalamus. But over the last 20 years, scientists have learned that almost all cell types—including fat cells, heart cells, and liver cells—have clock mechanisms, too. Increasingly, it seems these peripheral clocks can be entrained by (that is, changed to align with) environmental cues other than light, such as eating and activity. Moreover, exposure to these cues at times when they aren’t “expected” by the body may lead to obesity and related health effects.

For instance, in a study published in November 2009 in *Obesity*, PhD candidate Deanna Arble and colleagues from Northwestern University showed that mice fed during the day (when these nocturnal animals are normally asleep) gained a large amount of weight compared with control animals fed at night. The mice fed during the day did eat just a tiny amount more and moved a bit less than the control mice, which could have contributed to the weight gain. But the differences in activity and caloric intake were statistically insignificant, and it’s unlikely those factors were the sole cause of the weight gain because the two groups showed such drastic differences in body weight, says Arble.

In similar preliminary findings currently being written up for submission, Bray and Martin Young, an associate professor of cardiology also of the University of Alabama at Birmingham, found that rodents fed a high-fat diet at the end of their active phase gained more weight and had decreased glucose tolerance compared with animals that ate a high-fat diet at the beginning of their active phase but ate a protein-matched, low-fat control diet at the end of their active phase. “Evidence is beginning to accumulate to suggest that the time of day at which we consume not only total calories but also fat versus carbohydrates does quite profoundly influence how those calories are metabolized and therefore the risk of these metabolic diseases,” says Young.

In a human study published in the 17 March 2009 issue of *Proceedings of the National Academy of Sciences* by Frank A.J.L. Scheer and colleagues, symptoms of metabolic syndrome resulted when participants ate and slept at the wrong times—that is, out of alignment with their habitual circadian cycle. Over a 10-day experimental study, participants ate and slept at all phases of the circadian cycle. When they ate and slept about 12 hours out of phase from their habitual times, they showed decreased leptin levels, increased glucose (despite increased insulin), and increased mean arterial pressure. Three of the subjects showed post-meal glucose responses in the range typical of a prediabetic state.

Other research hints at the mechanisms involved in these links between disruption of circadian rhythms and metabolic syndrome. Using a mouse model in which heart-specific clock cells were disrupted, Young found these cells directly regulated triglyceride metabolism, as reported 25 November 2009 ahead of print in the *Journal of Biological Chemistry*. In beating hearts removed from normal mice—and thus free from hormonal and other factors in the body that influence triglyceride levels—Young observed normal fluctuation in the synthesis and breakdown of these esters throughout the day. But in hearts removed from heart-specific clock mutant mice, that oscillation was completely lost. “Those data showed that the cell-specific clock is regulating triglyceride metabolism,” Young says.

In another example, Jeff Gimble, a professor of stem cell biology at the Pennington Biomedical Research Center, has found evidence that levels of lipoprotein lipase, which prevents buildup of fats in the liver and arteries by moving them from the bloodstream into adipose tissue, oscillate throughout the day, peaking during mice’s active phase. Extrapolating from those data to speculate on what this may mean for humans, Grimble says, “If you’re eating fat when lipoprotein lipase is at its bottom level, you’re going to clear fats that much more slowly.”

## The Tip of the Iceberg?

Big questions still to be answered include which functions are regulated by the central clock, which are regulated by the peripheral clocks, and how the various clocks interact. It’s hard to separate out those questions in many of the classic mouse models, which are global knockouts of clock genes in all tissues. These models show very different phenotypes. For example, global loss of *Bmal1* leads to a mouse that tends to be lean but has complete loss of circadian clock function in almost every cell type, problems with glucose homeostasis, and a reduced life span. Mice with a mutated *Clock* gene are obese and have features of metabolic syndrome, but they show relatively normal activity levels under normal light/dark cycles and show abnormal behavior only in complete darkness.

Are these differences caused by the knockout of the central clock, the knockout of the peripheral clocks, the way activity and feeding changes are affecting peripheral clocks, or even the strain of mouse used? Researchers are beginning to address those questions with cell-specific models. For instance, by knocking out the clock only in fat cells in a mouse model, Bray and Young have induced metabolic syndrome in the animal. “Most of the phenotypes observed in the global clock knockout are also present in the adipocyte-specific model,” Bray says.

Some scientists also wonder whether the effects seen from disruption of clock genes are caused by the genes’ roles in circadian rhythms or by some other unknown functions of the genes. “This is a little controversial, but there are some data out there suggesting that some of these clock genes, such as *PER2*, have functions independent of the clock,” Young says. For example, a study from Rainer Spanagel and colleagues published in the January 2005 issue of *Nature Medicine* suggested *Per2* influenced alcohol consumption in mice via the neurotransmitter glutamate.

Because of such observations, some see very widespread ramifications of learning more about metabolism, obesity, and circadian clock functions. A leading researcher in circadian rhythm studies, Fred Turek of Northwestern University, has suggested the close relationship between metabolism and clock functions may just be the tip of the iceberg. Writing in the 18 December 2008 issue of *Nature*, he proposed the clock may be the “conductor of the orchestra” that keeps all the body’s behavioral and physiological functions working in harmony.

“I don’t think Dr. Turek is reaching by saying that,” Bray says. “We know that disruptions in the DNA sequence of some clock-related genes are associated with seasonal affective disorder and bipolar disorder. But how that works is just not known.”

## Figures and Tables

**Figure:**
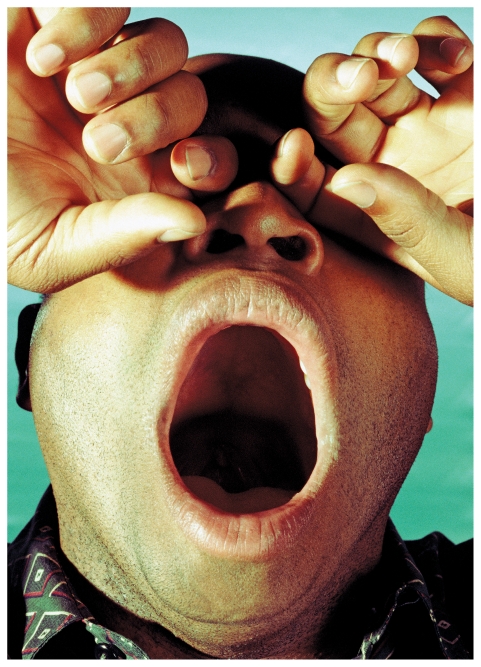


**Figure:**
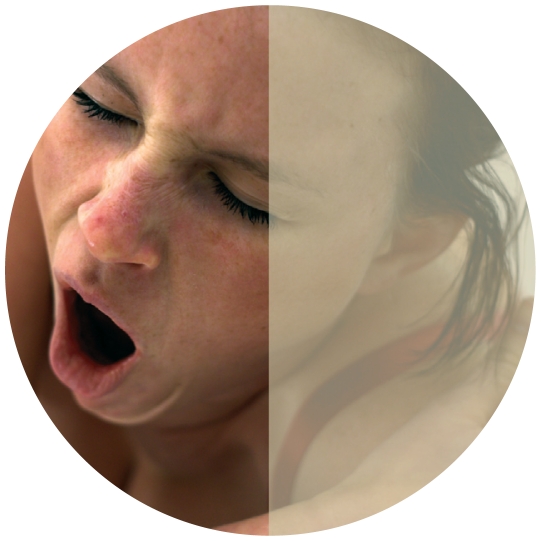
**“Biological night”** is defined as the period between the onset and cessation of melatonin secretion. During this period, melatonin is secreted, blood cortisol levels rise, core body temperature goes down, and we become sleepy. Melatonin is produced only during darkness and stops upon optic exposure to bright light, with light in the blue portion of the visible spectrum proving the most potent at suppressing production [for more information about circadian rhythm and blues, see “What’s in a Color? The Unique Human Health Effects of Blue Light,” p. A22 this issue].

**Figure:**
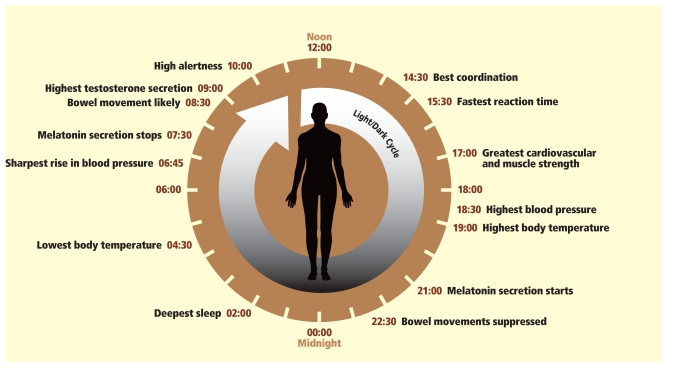
**The Circuit of a Day** **The word “circadian” derives from the Latin** ***circa*, meaning “approximately,” and** ***dies*, meaning “day.” The circadian clock (as shown here representing a person who rises early in the morning and sleeps at night) synchronizes with cycles of light/dark, eating, and activity.** Source: School of Biological Sciences, Royal Holloway University of London. Adapted by Matthew Ray/EHP.

**Figure:**
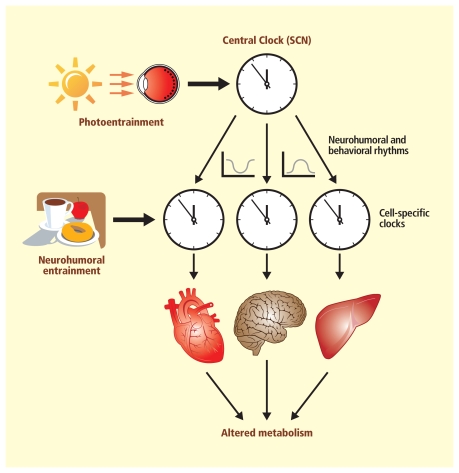
**One Body, Many Clocks** **Increasing evidence links disruptions in the body’s various circadian timekeepers to obesity and malfunctions in metabolism. It’s generally accepted that light exposure can reset the main clock in the suprachiasmatic nucleus of the brain, and that cues from the main clock as well as from eating and activity can reset peripheral clocks that operate in almost all the body’s cells.** Source: Bray MS, Young ME. 2009. The roll of cell-specific circadian clocks in metabolism and disease. Obes Rev 10(suppl. 2):6–13. Adapted by Matthew Ray/EHP.

**Figure:**
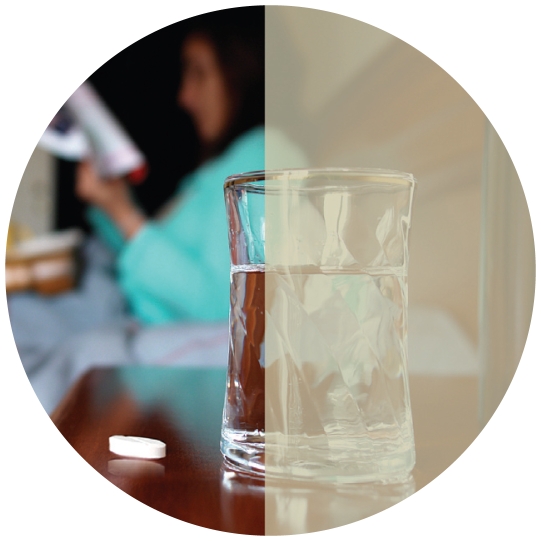
**As researchers learn more** about how metabolism and other functions are linked to circadian rhythm, some see the potential to identify small molecules that could be made into pharmaceuticals that would act on the core clock regulators to treat sleep disorders as well as obesity and related health effects. Others are looking at how changing the time when current drugs are taken may make them work better. For instance, in the September 2008 issue of The Journal of Pharmacology and Experimental Therapeutics, Richard R. Almon and colleagues wrote that taking cholesterol-lowering statins at bedtime, as is currently recommended, may be less than optimal given that HMG-CoA reductase, the target for these drugs, has a maximal expression at approximately 10:00 a.m.

**Figure:**
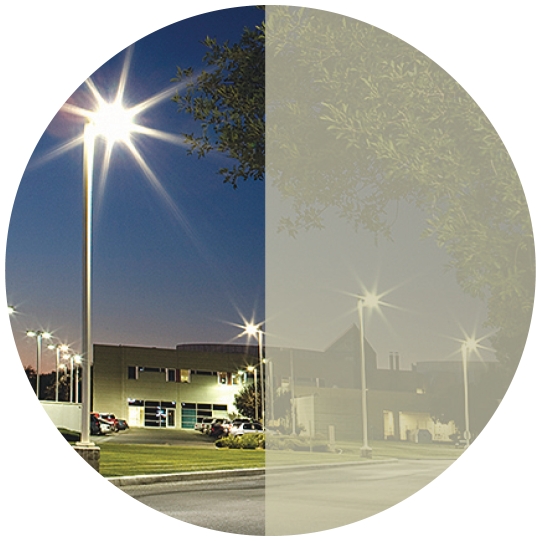
**Glare and other forms of light pollution** are recognized by the American Medical Association (AMA) as a public health issue of concern. On 15 June 2009 the AMA adopted a resolution to support the reduction of light pollution caused by outdoor artificial lighting, citing its implication in disrupting human and animal circadian rhythms as well as its “strongly suspected” role in suppressed melatonin production, depressed immunity, and increased rates of certain cancers. The AMA pledged in the resolution “to develop and enact a policy that supports light pollution reduction efforts and glare reduction efforts at both the national and state levels.”

